# An Investigation of Age-Related Iron Deposition Using Susceptibility Weighted Imaging

**DOI:** 10.1371/journal.pone.0050706

**Published:** 2012-11-30

**Authors:** Dan Wang, Wen-Bin Li, Xiao-Er Wei, Yue-Hua Li, Yong-Ming Dai

**Affiliations:** 1 Institute of Diagnostic and Interventional Radiology, The Sixth Affiliated People’s Hospital, Shanghai Jiao Tong University, Shanghai, People’s Republic of China; 2 Siemens MRI Technique Support Department, German Siemens Healthcare MR Clinical Application, Shanghai, People’s Republic of China; Stanford University School of Medicine, United States of America

## Abstract

**Aim:**

To quantify age-dependent iron deposition changes in healthy subjects using Susceptibility Weighted Imaging (SWI).

**Materials and Methods:**

In total, 143 healthy volunteers were enrolled. All underwent conventional MR and SWI sequences. Subjects were divided into eight groups according to age. Using phase images to quantify iron deposition in the head of the caudate nucleus and the lenticular nucleus, the angle radian value was calculated and compared between groups. ANOVA/Pearson correlation coefficient linear regression analysis and polynomial fitting were performed to analyze the relationship between iron deposition in the head of the caudate nucleus and lenticular nucleus with age.

**Results:**

Iron deposition in the lenticular nucleus increased in individuals aged up to 40 years, but did not change in those aged over 40 years once a peak had been reached. In the head of the caudate nucleus, iron deposition peaked at 60 years (*p*<0.05). The correlation coefficients for iron deposition in the L-head of the caudate nucleus, R-head of the caudate nucleus, L-lenticular nucleus and R-lenticular nucleus with age were 0.67691, 0.48585, 0.5228 and 0.5228 (*p*<0.001, respectively). Linear regression analyses showed a significant correlation between iron deposition levels in with age groups.

**Conclusions:**

Iron deposition in the lenticular nucleus was found to increase with age, reaching a plateau at 40 years. Iron deposition in the head of the caudate nucleus also increased with age, reaching a plateau at 60 years.

## Introduction

Excessive deposition of iron in brain may be a risk factor for degenerative disease [1], [2], [3] and knowledge of the normal range of iron accumulation is essential [4]. In particular, the lenticular nucleus may exhibit susceptibility to mineralization as a result of its high metabolic rate [5]. Excessive mineral deposition may, in turn, restrict blood flow and cause neural tissue injury that leads to further mineralization [4], [6], [7]. It is well documented that the iron content of the brain increases with age, particularly in the lenticular nucleus, and abnormal levels of iron in the central nervous system (CNS) are found in several neurodegenerative diseases [8], [9], [10]. Understanding the appearance of iron deposition in the aging brain is an important step in the interpretation of imaging of the diseased brain. In this study, the head of the caudate nucleus and lenticular nucleus were selected as the objects. Haacke et al. selected the same anatomical sites [4], [11], [12], [13], and illustrated the importance of establishing a brain iron deposition range in healthy volunteers in order to determine the pattern in diseased brains. However, although the globus pallidus and caudate nucleus were measured, differences according to age were not measured [4]. In this study, iron deposition was further analyzed in accordance with age-related changes.

Susceptibility Weighted Imaging (SWI) provides a new method for enhancing contrast in MR imaging. Conventional imaging that relies on the magnitude of information to generate the image and phase information has been discarded, with the exception of a few flow imaging applications. Phase images and the magnitude images can be combined to create susceptibility-weighted images [7]. In addition, phase images can be used to quantify iron accumulation [4], [14], [15]. The aim of this study was to quantify iron accumulation in the lenticular nucleus and the caudate nucleus of healthy volunteers of different ages using SWI.

## Materials and Methods

### Subjects

**Figure 1:**
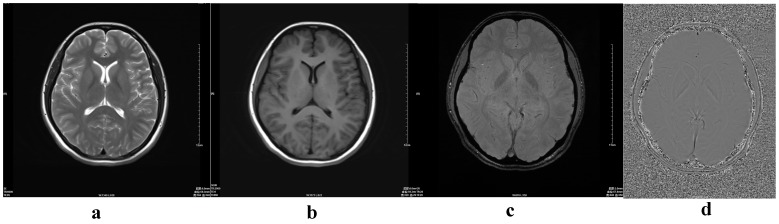
MRI findings in a 17-year-old normal girl. (a) axial T2-weighted image; (b) axial T1-weighted image; (c) magnitude image; (d) phase image.

**Figure 2:**
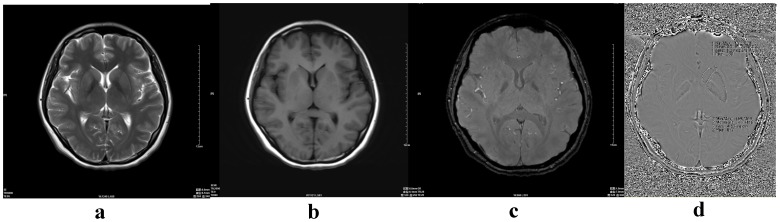
A 30-year-old normal female. (a) axial T2-weighted image; (b) axial T1-weighted image; (c) magnitude image; (d) phase image (including the measurement method ROI 1- head of the caudate nucleus, ROI 2- lenticular nucleus ).

**Figure 3:**
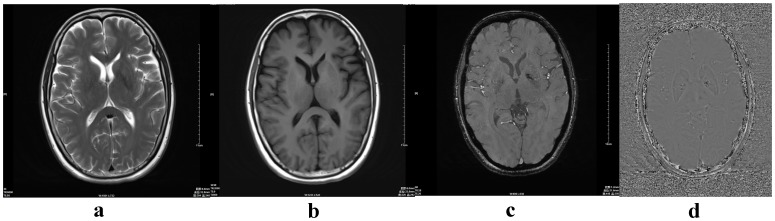
A 58-year-old normal male. (a) axial T2-weighted image; (b) axial T1-weighted image; (c) magnitude image; (d) phase image.

**Figure 4:**
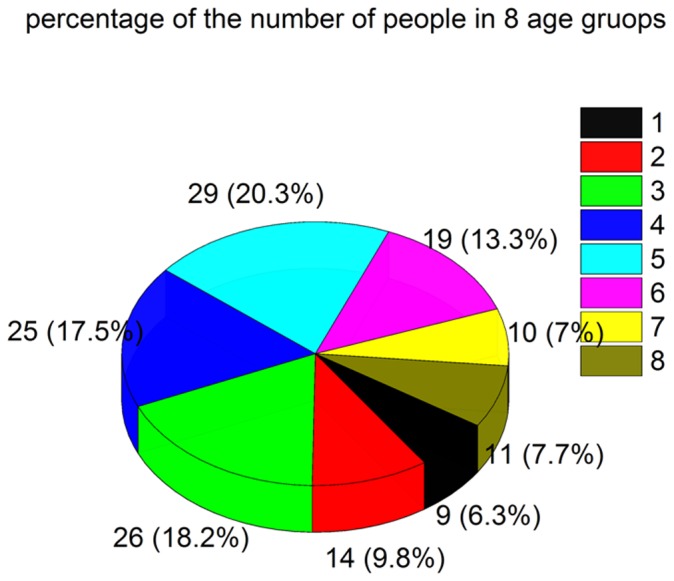
Numbers and percentages of individuals in each of the eight age groups of the total 143 subjects. group 1 (9, 6.3%), group 2 (14, 9.8%), group 3 (26,18.2%), group 4 (25,17.5%), group 5 (29,20.3%), group 6 (19,13.3%), group 7 (10,7%), and group 8 (11,7.7%).

We retrospectively selected 143 healthy volunteers (70 male, 73 female) who were imaged between June 2011 and November 2011 (Table 1). These volunteers ranged in age from 12 to 87 years (mean age, 48.8 years; SD, 18.8 years). Exclusion criteria included structural abnormalities that could produce dementia, such as cortical infarction, tumors, subdural hematoma, brain trauma, epilepsy, alcoholism, psychiatric illness, or other systemic diseases that may affect brain function. All volunteers were right-handed. They were divided into eight groups according to age: group 1 (10–19 years), group 2 (20–29), group 3 (30–39), group 4 (40–49), group 5 (50–59), group 6 (60–69), group 7 (70–79), and group 8 (80–89) (Fig. 1a–d,2a–d,3a–d,4). MR examinations included a normal conventional MR imaging examination (sagittal T1, axial T2, and axial fluid-attenuated inversion recovery, FLAIR sequences) and the acquisition of a normal SWI sequence.

We obtained written informed consent prior to the MR imaging examinations either from volunteers themselves or from their guardians, as relevant, for the use of their data. This study was reviewed and approved by the Ethics Review Board of the Shanghai 6^th^ People’s Hospital Affiliated with Shanghai Jiao Tong University.

### MR Examination and Measurements

A 3T MR Scanner (MAGENTOM, Verio, Siemens Healthcare, Erlangen, Germany) with a 32-channel head coil was used. The imaging sequences included conventional MR sequences (T1-weighted imaging, T2-weighted imaging)/DWI (diffusion weighted imaging)/SWI. Protocols were as follows: T2: FOV 250 mm, TR/TE 6,000/95 ms, flip angle 150°, matrix 384×384, slice thickness 6 mm, distance factor 30%; T1 FLAIR: FOV 250 mm TR/TE 2,000/9 ms, flip angle 150°, matrix 320×320, slice thickness 6 mm, distance factor 30%, SWI: FOV 230 mm TR/TE 28/20 ms, flip angle 15°, matrix 320×320, resolution 0.7×0.7×1.2 slice thickness 1.2 mm, distance factor 30%. A group of magnitude, phase, minimum intensity projection (MIP) and SWI images were automatically online reconstructed [4].

### Image Analysis

Phase images were used to quantify iron deposition. On the Siemens Workstation, two neuroradiologists (with 5 and 8 years of experience, respectively) manually outlined the lenticular nucleus and the head of the caudate nucleus as regions of interest (ROI). Following this, the mean phase values of the lenticular nucleus and the head of the caudate nucleus were measured by the following conversion formula.

As the measured value (Y) has a range of (−4096∼4095) which maps to the phase value×(Pi ∼ -Pi) (MAGNETOM, Verio, Software, VB17), so the conversion formula was presented as follows;

The values thus obtained were the head of the caudate nucleus and the lenticular nucleus inside the angles’ mean values in phase images. Following measurement, the mean values were calculated for statistical analysis. The measurement method can be seen in Fig. 2d. The ANOVA model was used to compare values for the head of the caudate nucleus or lenticular nucleus between the different age groups using the Fish-LSD test (two sample T test comparison). For correlation analysis, Pearson’s correlation coefficient was used to compare values for the iron deposition in head of the caudate nucleus or lenticular nucleus with age. Linear regression analysis and polynomial fitting were performed to compare the iron deposition in the head of the caudate nucleus or lenticular nucleus with age. In the linear analysis, if the *P*-value was less than 0.05, this indicated that a linear relationship existed between the iron deposition and age.

## Results

**Figure 5:**
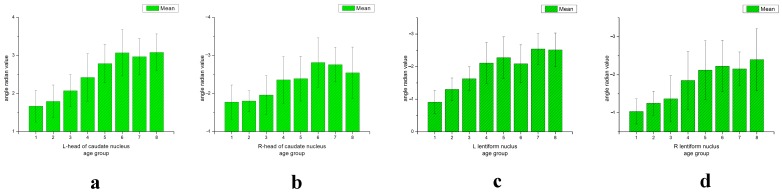
The mean angle radian value and standard deviation (SD) of the bilateral head of the caudate nucleus or bilateral lenticular nucleus of 8 age groups (a–d).

Iron deposition within the lenticular nucleus increased with age before 40 years (*P*<0.05), with groups 1 to 4 maintaining an upwards trend. Over 40 years, there was no significant increase in iron deposition. Iron deposition increased in the head of the caudate nucleus in individuals aged up to 60 years (*P*<0.05). It reached a peak at this age and did not change significantly in older individuals. In groups 1 to 6, a general upwards trend in iron deposition levels was observed (Fig. 5a–5d, 6a–7d, 7a–7d; Tables 1,2,3,4).

**Table 1:** Summary and comparison of iron deposition in L-head of caudate nucleus between 8 Age Groups.

Parameters	Statistics	Age Group 1	Age Group 2	Age Group 3	Age Group 4	Age Group 5	Age Group 6	Age Group 7	Age Group 8
L- head of caudate nucleus	Number	9	14	26	25	29	19	10	11
angle radian mean	Mean	−1.66831	−1.79653	−2.07587	−2.42381	−2.78912	−3.071	−2.96834	−3.08296
	STD	0.41493	0.42786	0.42333	0.62201	0.50547	0.60807	0.4776	0.48431
	Min	−1.14473	−1.10486	−1.27873	−1.34473	−1.49414	−1.90967	−2.10625	−2.54883
	Max	−2.61807	−2.53594	−3.04541	−3.44677	−3.51123	−4.27588	−3.45846	−3.81445
Comparison with Age Group 1	*P* value	–	0.56063#	0.04246*	<0.001*	<0.001*	<0.001*	<0.001*	<0.001*
Comparison with Age Group 2	*P* value	–	–	0.10376#	<0.001*	<0.001*	<0.001*	<0.001*	<0.0019*
Comparison with Age Group 3	*P*value	–	–	–	0.0171*	<0.001*	<0.001*	<0.001*	<0.001*
Comparison with Age Group 4	*P*value	–	–	–	–	0.01031*	<0.001*	0.00539*	<0.001*
Comparison with Age Group 5	*P* value	–	–	–	–	–	0.06558&	0.34384#	0.10908#
Comparison with Age Group 6	*P* value	–	–	–	–	–	–	0.61033#	0.95116#
Comparison with Age Group 7	*P* value	–	–	–	–	–	–	–	0.61093#

# *P*-value >0.1, mean that there was no significant difference between adjacent groups.

* *P*-value <0.05, mean that there was significant difference between adjacent groups.

& 0.1>*P*-value >0.05, mean that there was difference between adjacent groups, but the difference was not significant.

**Table 2:** Summary and comparison of iron deposition in R-head of caudate nucleus between 8 Age Groups.

Parameters	Statistics	Age Group 1	Age Group 2	Age Group 3	Age Group 4	Age Group 5	Age Group 6	Age Group 7	Age Group 8
R-head of caudate nucleus	Number	9	14	26	25	29	19	10	11
angle radian mean	Mean	−1.77686	−1.80411	−1.96309	−2.36131	−2.39327	−2.81603	−2.76012	−2.54643
	SD	0.44666	0.27505	0.50498	0.61057	0.58636	0.64712	0.45314	0.67395
	Min	−1.01953	−1.35767	−1.13818	−1.3667	−1.23486	−1.72266	−2.00391	−2.0083
	Max	−2.46973	−2.39667	−2.93994	−3.7749	−3.57715	−4.16162	−3.47561	−3.67383
Comparison with Age Group 1	*P* value	–	0.90851#	0.38641#	0.00753*	0.00416*	<0.001*	<0.001*	0.00243*
Comparison with Age Group 2	*P* value	–	–	0.38836#	0.0031*	0.00138*	<0.001*	<0.001*	0.00114*
Comparison with Age Group 3	*P* value	–	–	–	0.0114*	0.00471*	<0.001*	<0.001*	0.00402*
Comparison with Age Group 4	*P* value	–	–	–	–	0.83293#	0.00791*	0.05653&	0.35749#
Comparison with Age Group 5	*P* value	–	–	–	–	–	0.01081*	0.07328&	0.43645#
Comparison with Age Group 6	*P* value	–	–	–	–	–	–	0.7966#	0.20131#
Comparison with Age Group 7	*P* value	–	–	–	–	–	–	–	0.37906#

# *P*-value >0.1, mean that there was no significant difference between adjacent groups.

* *P*-value <0.05, mean that there was significant difference between adjacent groups.

& 0.1>*P*-value >0.05, mean that there was difference between adjacent groups, but the difference was not significant.

**Table 3:** Summary and comparison of iron deposition in L- lenticular nucleus between 8 Age Groups.

Parameters	Statistics	Age Group 1	Age Group 2	Age Group 3	Age Group 4	Age Group 5	Age Group 6	Age Group 7	Age Group 8
L-globus pallidus	Number	9	14	26	25	29	19	10	11
angle radian mean	Mean	−0.91125	−1.30242	−1.62939	−2.11149	−2.27867	−2.08752	−2.54132	−2.51487
	SD	0.36301	0.34917	0.36376	0.62207	0.63444	0.58073	0.48416	0.51618
	Min	−0.40645	−0.73127	−0.89209	−0.92142	−1.35352	−1.12939	−1.9248	−1.84131
	Max	−1.42715	−1.72705	−2.35321	−3.57471	−3.51641	−2.98828	−3.40825	−3.20801
Comparison withAge Group 1	*P* value	–	0.08402&	<0.001*	<0.001*	<0.001*	<0.001*	<0.001*	<0.001*
Comparison with Age Group 2	*P* value	–	–	0.06292&	<0.001*	<0.001*	<0.001*	<0.001*	<0.001*
Comparison with Age Group 3	*P* value	–	–	–	0.00136#	<0.001*	0.00455*	<0.001*	<0.001*
Comparison with Age Group 4	*P* value	–	–	–	–	0.24625#	0.8812#	0.03069#	0.03587*
Comparison with Age Group 5	*P* value	–	–	–	–	–	0.22037#	0.17557#	0.20692#
Comparison with Age Group 6	*P* value	–	–	–	–	–	–	0.02891*	0.03379*
Comparison with Age Group 7	*P* value	–	–	–	–	–	–	–	0.90855#

# *P*-value >0.1, mean that there was no significant difference between adjacent groups.

* *P*-value <0.05, mean that there was significant difference between adjacent groups.

& 0.1>*P*-value >0.05, mean that there was difference between adjacent groups, but the difference was not significant.

**Table 4:** Summary and comparison of iron deposition in R- lenticular nucleus between 8 Age Groups.

Parameters	Statistics	Age Group 1	Age Group 2	Age Group 3	Age Group 4	Age Group 5	Age Group 6	Age Group 7	Age Group 8
R-globus pallidus	Number	9	14	26	25	29	19	10	11
angle radian mean	Mean	−1.03417	−1.25206	−1.36727	−1.85215	−2.1232	−2.22671	−2.15491	−2.40025
	SD	0.33062	0.3141	0.60792	0.76526	0.77487	0.67572	0.44468	0.81648
	Min	−0.50313	−0.71745	−0.57643	−0.71631	−1.16016	−1.39746	−1.65853	−1.34268
	Max	−1.4502	−1.79307	−3.26475	−3.53233	−5.03008	−3.68262	−2.87021	−3.62988
Comparison with Age Group 1	*P* value	–	0.44151#	0.19458#	0.0018*	<0.001*	<0.001*	<0.001*	<0.001*
Comparison with Age Group 2	*P* value	–	–	0.59974#	0.00737*	<0.001*	<0.001*	0.00123*	<0.001*
Comparison with Age Group 3	*P* value	–	–	–	0.0098*	<0.001*	<0.001*	0.00169*	<0.001*
Comparison with Age Group 4	*P* value	–	–	–	–	0.13511#	0.06468&	0.2228#	0.0234*
Comparison with Age Group 5	*P* value	–	–	–	–	–	0.59644#	0.89606#	0.23841#
Comparison with Age Group 6	*P* value	–	–	–	–	–	–	0.78132#	0.48931#
Comparison with Age Group 7	*P* value	–	–	–	–	–	–	–	0.3969#

# *P*-value >0.1, mean that there was no significant difference between adjacent groups.

* *P*-value <0.05, mean that there was significant difference between adjacent groups.

& 0.1>*P*-value >0.05, mean that there was difference between adjacent groups, but the difference was not significant.

**Figure 6:**
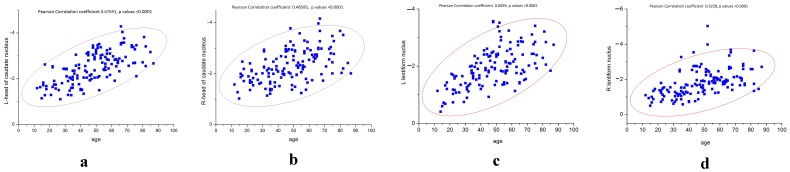
The Pearson’s correlations are shown between iron deposition in the L/R-head of the caudate nucleus or L/R-lenticular nucleus with age (a–d).

**Figure 7:**
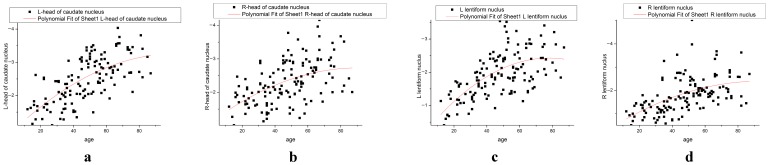
Iron deposition in the head of the caudate nucleus or lenticular nucleus correlated with age, using the polynomial fitting model (a–d). Iron deposition in the bilateral lenticular nucleus increased with age, reaching a plateau at 40 years. Iron deposition in the bilateral head of the caudate nucleus also increased with age, and reached a plateau at 60 years.

Differences in the angle radian values of the head of the caudate nucleus and the lenticular nucleus were compared between groups (Tables 1,2,3,4). It was found that the right lenticular nucleus angle radian values of approximately one quarter of volunteers was higher than those of the left lenticular nucleus, and this predominantly affected female volunteers (Fig. 5c–5d, Fig. 6c–6d, Fig. 7c–7d).

Correlations coefficients (r-values) for age versus iron deposition in the L-head of the caudate nucleus, R-head of the caudate nucleus, L-lenticular nucleus and R-lenticular nucleus were 0.67691, 0.48585, 0.5228 and 0.5228, respectively (*P*<0.001 for all). This suggests that iron deposition levels in both the head of the caudate nucleus and the lenticular nucleus were highly correlated with age (Fig. 6a–6d, Table 5). Linear regression analysis showed that the iron deposition level in both the R-head and L-head of the caudate nucleus, and the L-lenticular nucleus and R-lenticular nucleus had linear relationships with age (*P*<0.001 for all).

**Table 5:** Pearson Correlation coefficient of angle radian mean in head of caudate nucleus, lenticular nucleus and age.

	Number	Mean	SD	Min	Max	Pearson Correlation	P value
age	143	48.7972	18.787	12	87		
L-head of caudate nucleus	143	−2.50044	0.69331	−1.10486	−4.27588	0.676918	<0.001
R-head of caudate nucleus	143	−2.3066	0.64184	−1.01953	−4.16162	0.48585	<0.001
L- lenticular nucleus	143	−1.96089	0.68555	−0.40645	−3.57471	0.6059788	<0.001
R- lenticular nucleus	143	−1.82183	0.77364	−0.50313	−5.03008	0.5228	<0.001

*p* values <0.001. This suggests that both iron deposition in the head of the caudate nucleus and age, and the lenticular nucleus and age, were highly related with age.

## Discussion

Iron deposition increases with age in neural tissues, particularly in the lenticular nucleus and red nucleus. A non-invasive imaging method that could distinguish and quantify areas of iron deposition would improve our ability to assess and monitor individuals with iron over-deposition diseases [16], [17], [18]. It has been shown that abnormal iron deposition is related to a variety of neurodegenerative diseases, including Alzheimer’s disease (AD) and Parkinson’s disease (PD) [2], [19], [20]. Therefore, the measurement and quantitative analysis of iron deposition would be important in clinical situations [2].

In this study, it was found that iron deposition in the lenticular nucleus increased with age, but this relationship was not a simple positive correlation (Fig. 6a–6d, 7a–7d). Iron deposition increased in those aged between 10 and 40 years, but did not change significantly thereafter (Fig. 5c–5d). In the of the head of the caudate nucleus, iron deposition also increased with age, with depositions increasing more slowly in those over 60 years of age (Fig. 5a–5b). This result was similar to that reported by Aquino et al., who found that iron increased gradually with age in the lenticular nucleus in a non-linear manner [1]. Akhlaghpoor et al. used T2* research methods and reached a similar conclusion that as age increases the signal in the lenticular nucleus decreases, with a maximal value of iron deposition occurring around 60 years of age [5]. The study by Haacke et al. confirmed that iron deposition in the globus pallidus and caudate nucleus increased with age. However, they concluded that iron uptake only increased after the age of 40, which differed from our results [11]. In their study, individuals were randomly enrolled, and were divided into two groups aged 20–39 years and 40–69 years. In our current study, enrolled subjects were divided into eight groups of approximately 20 individuals, with a larger age range from 12 to 87 years. This study has a 10-year-old age segment. Therefore, the current study has a more reasonable age distribution. Furthermore, the 32-channel head coil used in this study provided high quality images.

With age, the iron deposition may be connected to a variety of factors, including reduced oxidative phosphorylation, oligodendrocyte dysfunction, decreased dopamine production and conversion, abnormal blood-brain barrier permeability, and excessive blood iron concentrations; however such theories have not yet been substantiated [5], [12].

In this study, SWI imaging was used to analyze iron content in the brain quantitatively, based on certain considerations. Firstly, the phase imaging sequence is an effective tool for the noninvasive assessment of iron content in the brain *in vivo* [15]. The basic principle of detection is based on the fact that iron deposition will change the homogeneity of the local magnetic field and produce a loss of the phase condition, which results in a reduction of the T2W * signal [11], [21]. This technique has a higher resolution and higher signal to noise ratio (SNR) characteristics compared to conventional T2WI or T2W* sequences [12], [22], [23]. Secondly, the phase imaging sequence provides three-dimensional thin-layer images. These images have a higher spatial resolution, and they can effectively distinguish between the various anatomical and morphological structures of the brain. Thus, phase imaging sequencing provides a reasonable method for the measurement of iron deposition [24].

This study differs from other studies in that SWI phase imaging was used to quantify iron deposition. In previous studies, a signal strength method was used to measure iron deposition, using T2W* low signal intensity image classification [11], [13]. Furthermore, this study used the head of the caudate nucleus and the lenticular nucleus inside angle mean values to quantify iron deposition in the phase images to analyze the relationship between increasing iron deposition and age. In this study, the right lenticular nucleus angle radian values of some volunteers was higher than those of the left lenticular nucleus; we hypothesize that gender, hormone levels and duration of education, amongst other factors, may contribute to this difference, but further studies are needed to confirm this [25].

### Study Limitations

Firstly, this study focused on the quantitative measurement of caudate and lenticular nucleus iron deposition in healthy volunteers. This study did not involve other brain regions with high levels of iron deposition, such as the red nucleus and substantia nigra. This was mainly because that there have already been many reports regarding iron deposition in the red nucleus and substantia nigra, and these areas are known to have high levels of iron deposition in pathological conditions, such as AD. Secondly, there was a smaller sample size in groups 1 and 2, which may explain the lack of significant findings between these groups. Thirdly, if the iron content is too high, the SWI sequence and analytic software may underestimate the iron content to some extent [4], [11].

### Conclusions

SWI can be used to quantify age-dependent iron deposition changes in the caudate and lenticular nuclei under normal circumstances. As age increases, iron deposition increases in the lenticular nuclei, reaching a plateau when the individual is approximately 40 years old. Iron deposition in the head of the caudate nucleus reaches a plateau when the individual is approximately 60 years old, after which there is no significant increase.
